# Evidence for an Association Between a pH-Dependent Potassium Channel, TWIK-1, and the Accuracy of Smooth Pursuit Eye Movements

**DOI:** 10.1167/iovs.65.8.24

**Published:** 2024-07-16

**Authors:** Gary Bargary, Jenny M. Bosten, Adam J. Lawrance-Owen, Patrick T. Goodbourn, John D. Mollon

**Affiliations:** 1 Department of Psychology, University of Cambridge, Downing Street, Cambridge, United Kingdom; 2 Department of Optometry and Visual Science, City University London, Northampton Square, London, United Kingdom; 3 School of Psychology, University of Sussex, Falmer, United Kingdom; 4 School of Psychological Sciences, Faculty of Medicine, Dentistry and Health Sciences, University of Melbourne, Victoria, Australia

**Keywords:** pursuit eye movements, KCNK1, TWIK-1, ion channel

## Abstract

**Purpose:**

Within the healthy population there is a large variation in the ability to perform smooth pursuit eye movements. Our purpose was to investigate the genetic and physiological bases for this variation.

**Methods:**

We carried out a whole-genome association study, recording smooth pursuit movements for 1040 healthy volunteers by infrared oculography. The primary phenotypic measure was root mean square error (RMSE) of eye position relative to target position. Secondary measures were pursuit gain, frequency of catch-up saccades, and frequency of anticipatory saccades. Ten percent of participants, chosen randomly, were tested twice, giving estimates of test-retest reliability.

**Results:**

No significant association was found with three genes previously identified as candidate genes for variation in smooth pursuit: *DRD3*, *COMT*, *NRG1.* A strong association (*P* = 3.55 × 10^−11^) was found between RMSE and chromosomal region 1q42.2. The most strongly associated marker (rs701232) lies in an intron of *KCNK1*, which encodes a two-pore-domain potassium ion channel TWIK-1 (or K2P1) that affects cell excitability. Each additional copy of the A allele decreased RMSE by 0.29 standard deviation. When a psychophysical test of visually perceived motion was used as a covariate in the regression analysis, the association with rs701232 did not weaken (*P* = 5.38 × 10^−^^12^).

**Conclusions:**

Variation in the sequence or the expression of the pH-dependent ion channel TWIK-1 is a likely source of variance in smooth pursuit. The variance associated with TWIK-1 appears not to arise from sensory mechanisms, because the use of a perceptual covariate left the association intact.

We share with our primate relatives the capacity to track a smoothly moving object with our gaze.^1^ This phylogenetically recent ability serves to stabilize the object on the fovea—a retinal region that is rich in cones and is coupled to a disproportionately large area of the visual cortex.

Although the neural pathways that underlie smooth pursuit overlap with those that control saccades and although the two systems necessarily collaborate during tracking,^2^^–^^4^ there is evidence for some functional independence between these two types of eye movement. Whereas saccades are primarily driven by position error, smooth-pursuit movements are driven by sensory signals that represent stimulus velocity,^5^^,^^6^ signals that possibly derive from low-level motion detectors in the initial stage and from high-level motion systems once tracking is on target.^7^ Barbiturate drugs have a disproportionate effect on smooth pursuit: under the influence of barbiturates, tracking tasks are performed by a succession of saccades.^5^ Conversely, in some cases of idiopathic ocular motor apraxia, smooth pursuit movements may survive when horizontal saccadic movements are lost.^8^^,^^9^

Smooth pursuit eye movements are impaired, often disproportionately, in several other neurological and psychiatric conditions, including episodic ataxia type 4, Joubert syndrome, Alzheimer disease, and posterior cortical atrophy.^10^^–^^13^ The impairment of smooth pursuit in cases of psychosis is long established,^14^^–^^17^ although, in a healthy German population, Coors et al*.*^18^ found no consistent relationship between polygenic risk scores for schizophrenia and either the gain of smooth pursuit or the number of saccades made while the participant was tracking.

Within the non-clinical population, there are, however, large individual differences in smooth pursuit performance,^19^^,^^20^ and twin studies suggest that the accuracy of smooth pursuit is substantially heritable.^21^^,^^22^ Two early candidate-gene studies were prompted by the dopaminergic theory of schizophrenia. Thus Rybakowski and colleagues^23^ reported an association with the Ser-9-Gly polymorphism of the *DRD3* gene, encoding a dopaminergic receptor: The Ser-Ser phenotype was more likely to be accompanied by impaired pursuit, both in healthy controls and in patients with schizophrenia. Similarly, Thaker et al*.*^24^ reported that the Val-158-Met polymorphism of *COMT* was associated with differences in predictive pursuit gain in healthy individuals. Another gene of interest—again on account of its being a candidate gene for schizophrenia—has been *NRG1*, which encodes neuregulin-1. For a large sample of male military conscripts, Smyrnis and colleagues^25^ reported an association between root-mean-square error in smooth pursuit and the single nucleotide polymorphism (SNP) rs6994992 (SNP8NRG243177) in the promoter region of *NRG1*. However, negative results for this and other *NRG1* polymorphisms were reported for a Korean population^26^ and for an Icelandic population.^27^ In a whole-genome study of a mixed cohort of healthy controls and patients with psychotic conditions, Lencer and colleagues^28^ found no SNP with genome-wide significance for smooth pursuit gain but reported an association of *IPO8* (chromosome 12p11.21) with initial pursuit acceleration.

We describe here a whole-genome association study of smooth pursuit in healthy young adults. Many whole-genome association studies offer no formal measure of the test-retest reliability of the phenotypic measurements. Any day-to-day variation in participants or in the measurement procedures will reduce reliability, and this will set an upper limit to any genomic associations that can be obtained.^29^ In the present study, therefore, we designed the phenotypic measurements to achieve high test-retest reliability, and we report explicit values. Our study did not confirm associations reported in the candidate-gene literature (see above), but individual variations in accuracy of smooth pursuit were strongly associated with markers within the gene *KCNK1*, which encodes the two-pore-domain potassium channel known as TWIK-1 or K2P1.^30^^–^^32^

## Methods

### Participants

Oculomotor measures were recorded as part of the PERGENIC project, in which we tested a population of 1058 young adults (413 male) on a 2.5-hour battery of optometric, perceptual and oculomotor tests.^33^^–^^35^ Participants were recruited from the Cambridge area by advertisements within the University and online, and a large proportion were students at the University of Cambridge. Their age range was 16–40, with a mean age of 22.14 (standard deviation [SD] = 4.09). To reduce population stratification in our sample, participants were all of European descent, as established by the reported nationality of their four grandparents and by direct checks on genotypes. To establish test-retest reliabilities, we asked 10% of participants, randomly selected, to perform the test battery on a second occasion.

The study received approval from the Cambridge Psychology Research Ethics Committee. All participants gave written consent after having been given information about the study.

### Phenotypic Measures

Measurements of smooth pursuit were available for 1040 participants and for 103 of those participants tested twice. For the latter group, in all but three cases, the two testing sessions were at least one week apart: the range of intervals was 103 days and the median was 18.8 days (SD = 23.3 days).

Eye movements and head movements were recorded using the head-mounted JAZZ-novo system (Ober Consulting, Poznan, Poland), which samples at 1 kHz and records horizontal and vertical rotations of the eye using infrared oculography. The output signal represents the average of the two eyes. The noise level (along the horizontal axis) is equivalent to six minutes of visual angle. A chin-rest was used to minimize head movements and to maintain a viewing distance of 60 cm.

Stimuli were presented on a GDM-F520 CRT monitor (Sony, Tokyo, Japan) controlled by a VSG 2/5 graphics card (Cambridge Research Systems, Rochester, UK). The monitor had a refresh rate of 100 Hz and was synchronized with the JAZZ-novo by means of the independent timer present on the VSG card. The synchronization, tested empirically, was accurate to 1 ms.

The target was a white disk (diameter of 0.3°; luminance of 75 cd/m^2^) and was presented on a gray background (25 cd/m^2^). A smooth-pursuit trial began with the target located centrally for a duration chosen randomly from the range 500 to 1500 ms. The target then moved horizontally (to the left or to the right) at a constant speed (10°/s, 20°/s, or 30°/s) until it reached an eccentricity of 15°, whereupon it changed direction and moved to the opposite side of the screen, continuing this triangular waveform for 5.5 cycles. There were eight trials for each target speed. Participants were instructed to fixate the target at all times.

To maximize the reliability of the measurements, a spatial calibration was performed at regular intervals during each recording session: the participant was asked to fixate stationary targets (duration 1000 ms) at 15°, 10°, 5°, 0°, −5°, −10°, and −15° relative to the central fixation point. The gain and offset were calculated for each calibration using linear regression of the oculographic signal against the target values; and these factors were applied to the eye-movement data recorded following the calibration.

In the analysis of the oculomotor data, a saccade was detected if the eye acceleration exceeded a relative threshold value (six times the median value of the standard deviation of the acceleration signal during the first 80 ms of all trials for a particular participant). As the primary, global measure of tracking performance, we calculated the *root mean square error* (RMSE) of eye position relative to target position in degrees of visual angle. The complete pursuit signal was used excluding blinks. We also extracted three secondary measures: *Pursuit gain,* defined as eye velocity divided by target velocity after removal of saccades and blinks, and excluding regions where the target changed direction (i.e. regions where the eccentricity of the target was >10°); *Frequency of catch-up saccades* (defined as saccades in the direction of pursuit that decreased positional error); and *Frequency of anticipatory saccades* (defined as saccades in the pursuit direction that increased positional error and were >1.5° in amplitude^36^). Results for saccades are expressed as average number per second. The secondary phenotypic measures are not, of course, independent of the primary measure, RMSE.

### Genotyping

DNA was collected from saliva samples taken during the participants’ visits, using Oragene OG-500 kits (DNA Genotek Inc, Ottawa, Canada). DNA extraction and microarray processing were performed by Cambridge Genomic Services (University of Cambridge, UK) according to manufacturers’ protocols. A total of 1008 individuals were genotyped at 733,202 SNPs on the Illumina HumanOmniExpress BeadChip. Genotype calling was by custom clustering using the algorithm GenCall implemented in Illumina GenomeStudio. Twenty-eight individuals were excluded from the analysis, on the basis of genetic and phenotypic quality control. Criteria for exclusion were as follows: Inadequate eye-movement data (eight individuals), genotypic sex anomalies (three individuals), low (<0.97) genotyping call rate (one individual), population outliers (one individual) and duplicate or related samples (15 individuals). This left 980 individuals in the analysis (599 female). Quality control was also conducted on individual SNPs. Markers with >2% missing genotypes (12706 SNPs) and markers with <1% minor allele frequency (77,738 SNPs) were excluded, leaving 642,758 SNPs in the analysis.

### Statistical Analysis

Association analysis was conducted using PLINK (v. 1.07),^37^ assuming an additive genetic effect. To control for any residual population stratification resulting from multiple genetic subgroups or genetic admixture in our population, we used EIGENSOFT (v. 4.2)^38^ to extract the top three principal components (PCAs) of genetic variation in the sample. The three PCA axes were entered together with sex as covariates in the regression model. At any suggestive (*p* < 1 × 10^−5^) loci, 2.5Mb regions centered on these locations were defined for imputation. These regions were imputed using IMPUTE2 (v. 2.3.0)^39^^,^^40^ with the phased haplotypes of the 1000 genomes project.^41^ Association analysis of these high-density regions was then carried out on the genotype probabilities using the dosage association feature of PLINK, with the four covariates added to the regression model as before.

We also used a permutation test to verify potential associations.^42^ This method generates empirically derived null distributions and accounts for multiple testing across the genome. It is particularly useful for testing associations where assumptions of parametric tests may be violated. Phenotypic scores were randomly permuted within the cohort to provide a new set of genotype-phenotype pairings sampled under the null hypothesis. Linear regressions were then calculated at each SNP for each permutation. To account for residual stratification, we allowed permutation of phenotypic values only within population groups; these were defined using PLINK's clustering method, which uses complete linkage agglomerative clustering, based on pairwise identity-by-state distance.^37^ This method grouped our cohort into 11 clusters. A *P* value was calculated for a given SNP as the probability that the *P* value for that SNP in the original analysis was larger than the *P* value for any SNP over 10,000 permutations.

Finally, regions corresponding to the association signal were defined. These regions are blocks that are in linkage disequilibrium with the most strongly associated marker and contain other “clumped” SNPs that are associated with the phenotype below a specified *P* value. The range therefore defines the region likely to contain the gene of interest, where the causal polymorphism associated with the phenotype lies. We used PLINK's clumping function to define the regions, using a significance threshold of index SNPs of 0.00001, a significance threshold for clumped SNPs of 0.01, an LD threshold for clumping of 0.1 and a physical distance threshold for clumping of 1250Kb. For all significant or suggestive SNPs, cluster plots were inspected manually and genotype distributions were evaluated for deviation from Hardy-Weinberg equilibrium. All genomic references are based on NCBI Build 37.

### Using Performance on a Phenotypic Perceptual Task as a Covariate

For the participants in our present cohort, we hold measurements of visual thresholds for detecting coherent motion in an array of moving dots^43^: Thresholds were expressed as the proportion of dots that must be in coherent motion for the predominant direction of motion to be correctly reported. In the present GWAS of ocular tracking, we used participants’ performance on the coherent motion test as a covariate, to test to whether the phenotypic variance associated with *KCNK1* was of perceptual origin.

## Results

### Phenotypic Measures

**Figure 1.**
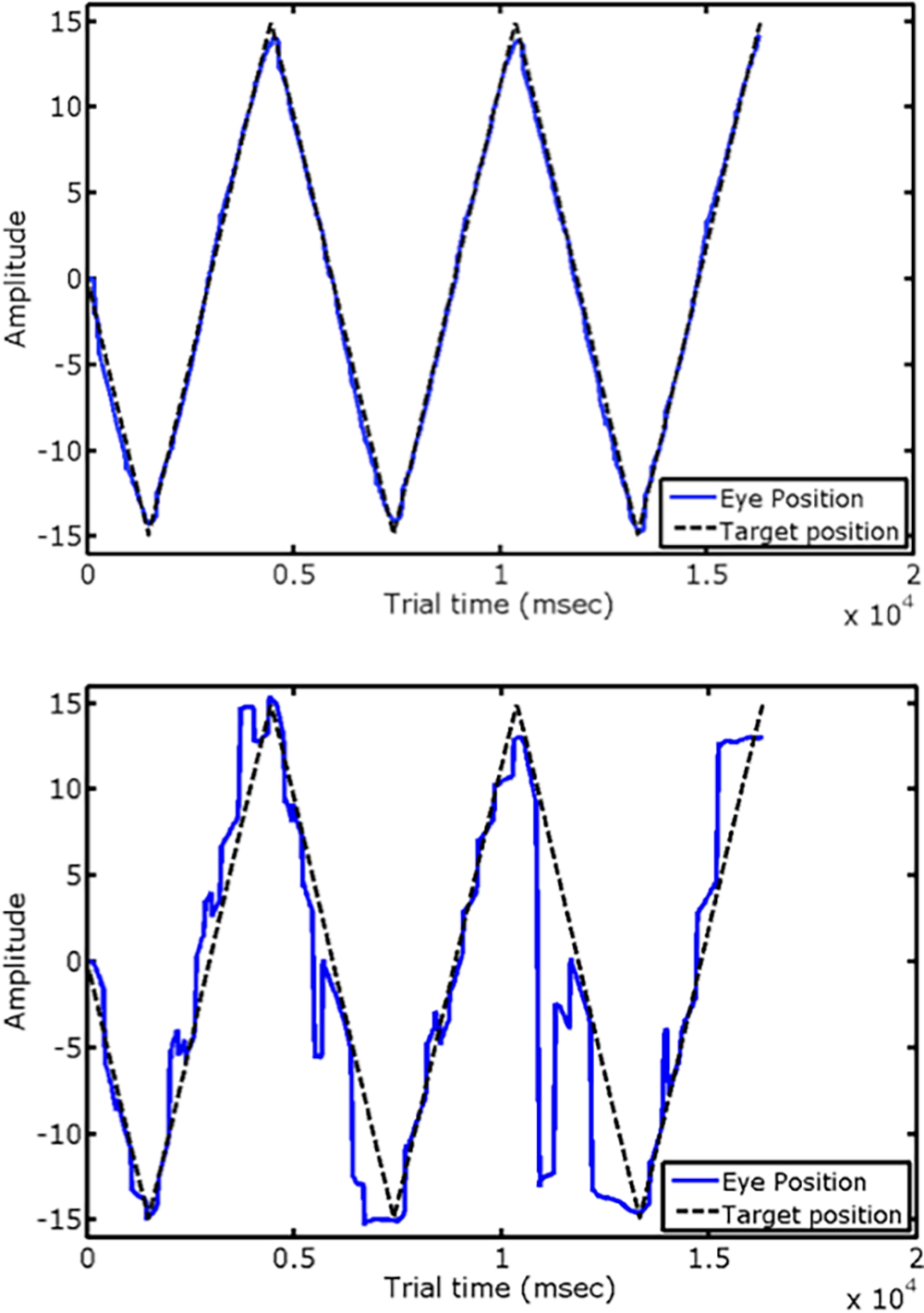
Examples of eye-movement records from participants who exhibited very good (above) and very poor (below) tracking performance in the smooth-pursuit task. The *broken line* represents the position of the target, which moves horizontally according to a triangular temporal waveform. The participant for whom a sample record is shown in the *upper panel* achieved an overall RMSE of 0.507 degrees of visual angle. The corresponding value was 5.98 for the participant whose sample is shown in the *lower panel*.

Within the cohort there were substantial individual differences in smooth-pursuit ability: Figure 1 shows examples of records from participants with very low and very high scores for the primary phenotypic measure, RMSE. Distributions for our phenotypic measures can be found in the study by Bargary et al*.*^20^ High test-retest reliabilities were found for RMSE and for the secondary measures—pursuit gain, anticipatory saccades, catch-up saccades. These values are shown in bold in Table 1. Also shown in Table 1 are the correlations between the phenotypic measures, which are in the expected directions. The values for test-retest reliability are based on the 10% of participants who were tested twice (*N* = 103), and the correlations between measures are based on the full cohort who completed the phenotypic tests (*N* = 1040). We give Spearman rank correlations, since the measures are not normally distributed. The values shown here are extracted from Tables 1 and 3 of Bargary et al*.*^20^

**Table 1.** Test-Retest Reliabilities (N = 103) of the Ocular Motor Measures (in Bold) and the Correlations of the Measures With Each Other for the Full Cohort (N = 1040)

	RMSE	Pursuit Gain	Catch-Up Saccades	Anticipatory Saccades
RMSE	**0.79**	−0.75	−0.41	0.71
Pursuit gain		**0.88**	0.28	−0.76
Catch-up saccades			**0.78**	−0.44
Anticipatory saccades				**0.83**

Values given are Spearman's rank-order correlation coefficients.

### Genetic Associations

Our array included three SNPs, rs6280 (*DRD3*), rs4680 (*COMT*) and rs6994992 (*NRG1*), that have been associated with smooth-pursuit performance in candidate-gene studies of healthy participants (see Introduction). We found no significant association between RMSE and any of these SNPs: for rs6280 the unadjusted *P* value was 0.067, for rs4680 it was 0.27, and for rs6994992 it was 0.108. These values were 0.121, 0.258 and 0.125, respectively, when performance on the perceptual coherent motion test was used as a covariate.

At the suggestion of a reviewer, we also asked whether our measure of smooth pursuit RMSE was significantly associated with any of the SNPs that reached genome-wide significance in the COGENT study of general cognitive ability.^44^ Only six of the 122 significant COGENT SNPs were directly available on our Illumina BeadChip array but we were able to impute all but two of the remainder (17:44366572:A:G and 17:44364573:G:A were not available). An association run in PLINK (with sex and the first three genetic PCAs as covariates) showed no significant associations (*P* > 0.099).

**Figure 2.**
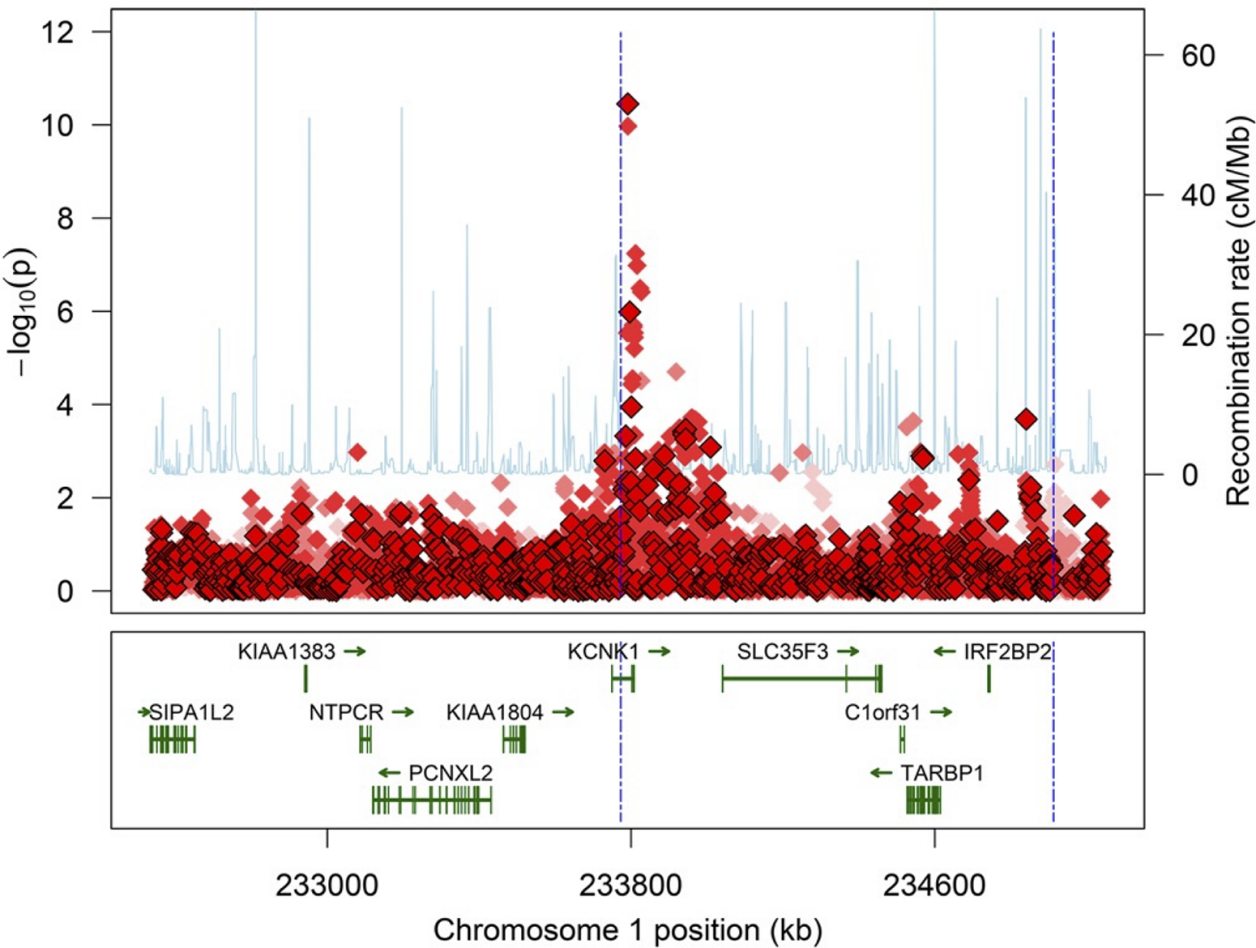
Manhattan plot of the association region. Measured SNPs are identified by *outline diamonds*, and imputed SNPs are without outlines. Saturation indicates imputation quality. Recombination rate is plotted with *solid blue lines*. The *vertical blue dashed lines* indicate the region identified by clustering, in which the critical variant is likely to lie. The genomic context of the region is shown below. *Vertical rectangles* indicate exons.

A strong genetic association was found between RMSE for smooth pursuit and a locus in the chromosomal region 1q42.2 that includes the gene *KCNK1* (Fig. 2). The most strongly associated genotyped SNP was rs701232 (*P* = 3.55 × 10^−11^) and the most strongly associated imputed SNP was rs701233 (*P* = 1.06 × 10^−10^). Both SNPs are located in the first intron of *KCNK1*, within a cluster of transcription-factor binding sites. The SNP rs701232 showed associations at the 1.7 × 10^−5^ and 8.0 × 10^−5^ levels with number of anticipatory saccades and with pursuit gain, and these associations disappeared when RMSE was included as a covariate (*P* = 0.47 for anticipatory saccades; *P* = 0.48 for gain). Interestingly, there was not a strong relationship with the frequency of catch-up saccades (*P* = 0.023). Table 2 lists all genotyped and imputed SNPs that lie on 1q42.2 and that have *P* values smaller than 5 × 10^−7^ for an association with RMSE.

**Table 2.** Association Results for the SNPs With *P* < 5 × 10^−7^

SNP	Location	LD	Allele 1	Allele 2	MAF	HWE *P* Value	β	SE	*P* Value
Genotyped									
rs701232	233791469	1	A	G	0.49	0.57	−0.53	0.08	3.55 × 10^−11^
Imputed									
rs701233	233791651	0.973	A	G	0.50	0.37	−0.52	0.08	1.24 × 10^−10^
rs12139277	233811896	0.267	A	C	0.24	0.38	−0.51	0.09	2.73 × 10^−8^
rs2884332	233815886	0.264	T	C	0.24	0.43	−0.50	0.09	6.36 × 10^−7^
rs143752646	233823693	0.228	A	C	0.26	0.28	−0.46	0.09	2.05 × 10^−7^
rs1039126	233827013	0.227	C	T	0.26	0.28	−0.46	0.09	3.05 × 10^−7^

β, change in RMSE per additional minor allele; HWE, Hardy-Weinberg equilibrium; MAF, minor allele frequency; SE, standard error of β.

All SNPs are located on chromosome 1. Locations are GRCh37 coordinates. LD is the *r*^2^ linkage disequilibrium between each SNP and rs701232. Allele 1 is the minor allele. All imputed SNPs have an IMPUTE2 quality score ≈ 1.

**Figure 3.**
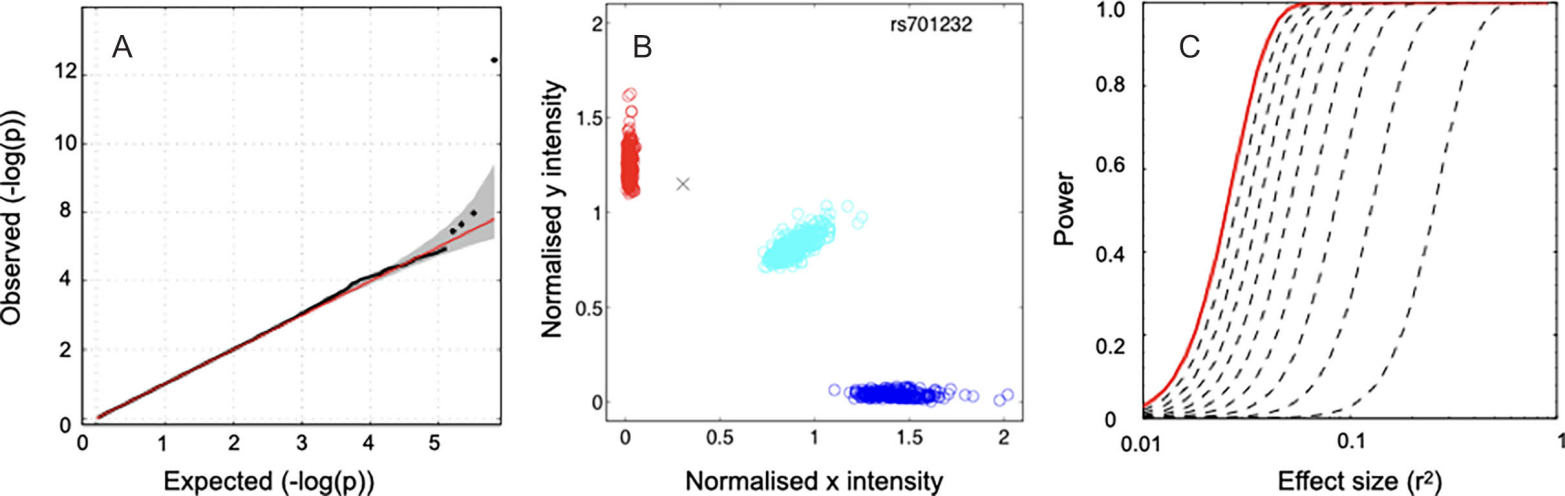
(**A**) Quantile-quantile plot of the *P* values resulting from the association test (black circles *P* < × 10^−5^; black line *P* > × 10^−5^). The null distribution is illustrated with the red line. The 95% confidence intervals are shown in *gray*. (**B**) Cluster plot for the genotyped SNP rs701232. Individuals included in the analysis are represented by *circles*, excluded individuals are represented by *crosses*. AA homozygous genotypes are *blue*, AB heterozygous genotypes are *cyan*, and BB homozygous genotypes are *red*. (**C**) Power to detect associations with *P* < 5 × 10^−7^. Effect size is the coefficient of determination (*r*^2^). The *red line* is for the case where the causal variant is in perfect LD (*r*^2^ = 1) with a genotyped SNP. The *dashed lines* illustrate the effect of reduced LD between any genotyped SNP and the causal variant. Power was calculated as follows: power = 1− *FF* (*Fcrit*|*ν1,ν2,λ*) where *FF* (⋅ |*ν1,ν2,λ*) represents the cumulative distribution function of the noncentral F distribution and *Fcrit* is the 100(1 − α) percentile from a central *F* distribution with *ν1* and *ν2* degrees of freedom and α is the α-level. *λ* is the noncentrality parameter *λ* = [*r*^2^/(1 – *r*^2^)] *ν2*. *ν1* was equal to 1, *ν2* was equal to 977, and α was equal to 5 × 10^−7^. The effect size of rs701232 is 0.043, and our power to detect an effect of this magnitude is 95%.

The quantile-quantile plot for the analysis (Fig. 3A) and the value of the genomic inflation factor (λ = 1.00) showed no evidence of increased signals due to technical error or to population stratification. Post-association quality control showed no evidence of departure from Hardy-Weinberg equilibrium (Table 2) and inspection of the signal intensity plots shows that the SNPs were well called (Fig 3B).

The minor allele frequency for the most strongly associated SNP, rs701232, was 0.49 in our sample, which is similar to the values of 0.46 recorded for the 1000 genome project and of 0.49 recorded for the GnomAD database. Each additional copy of the minority A allele at this position was associated with a decrease in RMSE equivalent to 0.29 standard deviation. The power to detect an effect of this magnitude was 95% (Fig. 3C).

Since the phenotypic data are not normally distributed,^20^ we also conducted the regression analysis using rank orders: The strongest signal was again at rs701232 (*P* = 7.74 × 10^−10^). Using the permutation method, which derives significance values without making assumptions about the distribution of the dataset, we again obtained the strongest signal at rs701232, with a genome-wide multiply-corrected *P* value of 0.0039.

### Using Coherent Motion Performance as a Covariate

There are moderate, but highly significant, phenotypic correlations between performance on our coherent motion test^43^ and the present oculomotor tracking measures. The values of Spearman's rho for the correlations of motion sensitivity with pursuit RMSE, with pursuit gain, with frequency of anticipatory saccades and with frequency of catch-up saccades were −0.28, 0.24, −0.22, and 0.14, respectively (*P* << 0.001 in all cases). Thus approximately 8% of the phenotypic variance is common to motion thresholds and to the RMSE of ocular tracking.

However, the marker rs701232—strongly associated with RMSE in oculomotor tracking—shows no sign of association with psychophysical sensitivity for coherent motion (uncorrected *P* = 0.70). We repeated our association analysis for oculomotor tracking, adding coherent motion sensitivity to the covariates previously used (sex and the first three PCAs of the genetic variation in our sample). The association of RMSE with rs701232 became somewhat stronger (*P* = −5.38 × 10^−12^) rather than weaker.

### Sex Differences

Our phenotypic analysis showed large sex differences in smooth-pursuit measures (see Table 2 of reference 20): Females showed an 18% higher mean RMS error than did males; their pursuit gain was lower by 4%; they made 30% fewer catch-up saccades (i.e., saccades that reduce the positional error); and they made 18% more anticipatory saccades (i.e., saccades that increase the positional error). Anticipatory saccades are often considered to be predictive and to be produced mistakenly in an attempt to improve tracking.^45^

**Figure 4.**
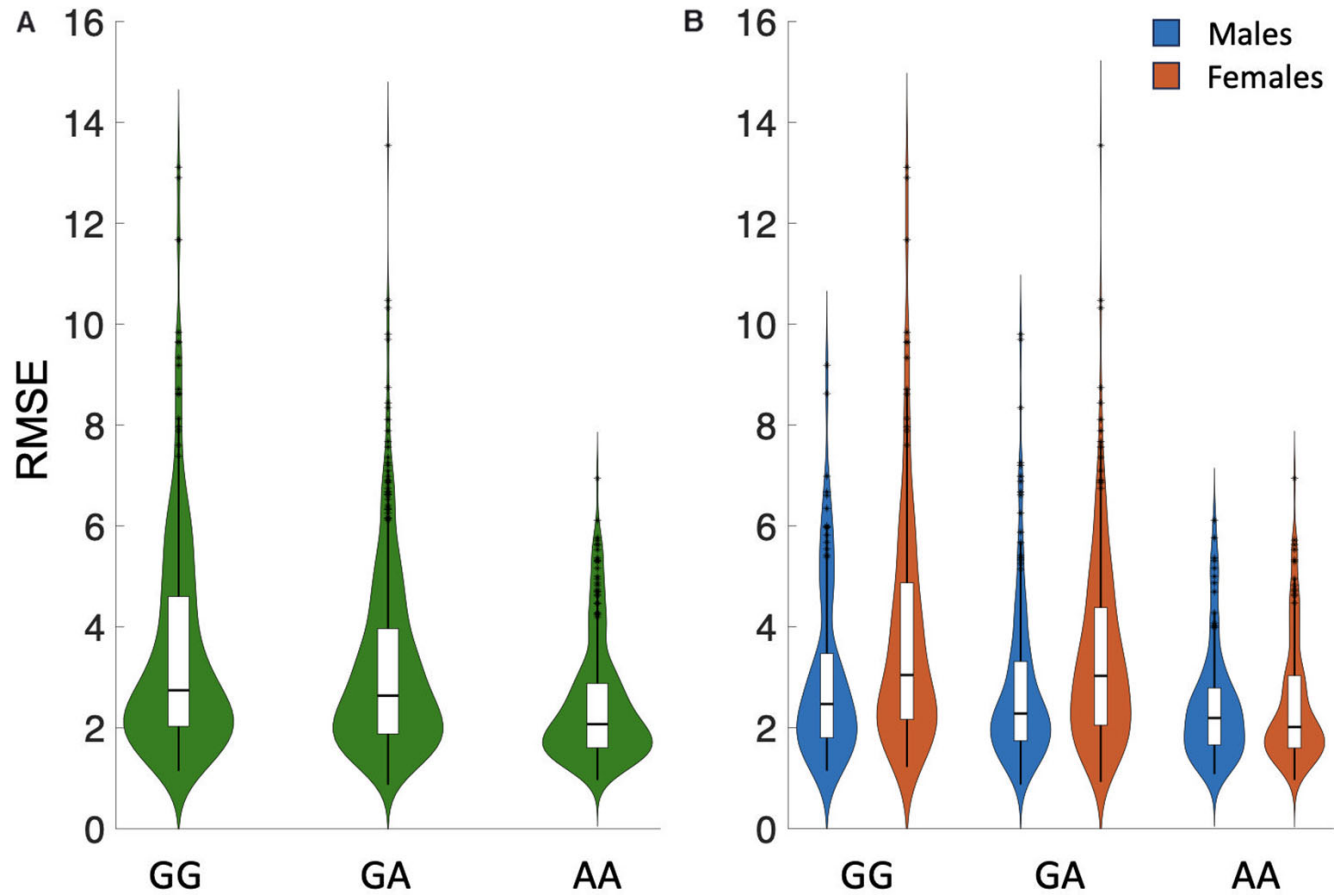
Distributions of RMSE by genotype. Violin plots with embedded box plots showing the distribution of pursuit RMSE by genotype at rs701232 (**A**) for the full sample, (**B**) separately for males and females.

These large phenotypic differences prompted us to examine the genetic associations of *KCNK1* separately for males and females (see Fig. 4 for violin plots by genotype). The association of rs701232 with RMSE remained very significant within the female cohort alone (*N* = 599; β = −0.69; *P* = 4.30 × 10^−10^), but in males the association was much weaker (*N* = 388; β = −0.28; *p* = 0.010). A permutation analysis showed that the effect size was significantly different between males and females: Across 10,000 permutations (where the full sample was split randomly into two cohorts of 388 and 592 to match the numbers of males and females), the probability that a difference in effect size was larger than the observed one was 0.005. The difference was not due to a difference in phenotypic reliabilities: None of the four phenotypic measures of smooth pursuit exhibited a significant sex difference in reliability within the 101 participants (61 female) who performed the measurements twice and whose genetic data were included in our analysis.

## Discussion

### Phenotypic Reliabilities

The strength of any association found in GWAS must depend on the test-retest reliability of the phenotypic measure, because some variance will always be either within-individual or instrumental in its origin^29^: Ideally the measure should wholly represent trait rather than state. Curiously, the reliability of the phenotypic measure is seldom stated in whole-genome studies of behavioral traits. In the case of the present study, we believe that the high reliabilities are the result of repeated calibration during the oculomotor testing of each participant.^20^

### Genetic Association

It is perhaps the high reliabilities of our phenotypic measures that allowed the emergence of a strong association between smooth-pursuit tracking performance and the gene *KCNK1* in chromosomal region 1q42.2. The size of the effect is relatively large: 0.29 SD for each additional copy of the A allele. Because we do not have a replication cohort and do not have the resources to carry out further testing, the association must remain provisional.

However, *KCNK1* encodes an ion channel and is a plausible candidate gene for an effect on smooth pursuit. It is widely expressed in the brain, both in neurons and in astrocytes. There are high levels of expression in the cerebellar granular cell layer, in the thalamic reticular nucleus, in the medial habenula and in the piriform cortex.^46^^,^^47^ In the neocortex, it is expressed most highly in layers 2/3 of the motor and frontal cortices.

The encoded protein, TWIK-1 or K2P1, is a two-pore-domain potassium ion channel (“Tandem of P-domains in a Weakly Inward rectifying K^+^ channel”).^30^^,^^31^ The channel itself is a dimer, assembled either from two units of TWIK-1 or a combination of TWIK-1 and another member of the two-pore-domain family, such as TREK-1, TASK-1, or TASK-3.^48^^,^^49^ The TWIK-1/TREK-1 heterodimer is common in astrocytes, where it mediates the K^+^ current responsible for background passive conductance but also mediates the release of glutamate from the cell when the heterodimer is bound to the G-protein subunit GNG4 as a result of activation of the heptahelical receptor, cannabinoid receptor 1.^50^^,^^51^

### Previous Associations of *KCNK1* with Pathologies

An early linkage study of a Mennonite kindred found that a form of episodic ataxia was associated with the 1q42.2 region containing *KCNK1*^52^: Disorders of this type are characterized by episodes of cerebellar dysfunction, and they typically arise from an inherited defect of an ion channel. Sequencing of the exons of *KCNK1* and adjacent splice sites in this family did not reveal mutations but did not rule out variants that could change the expression of the gene. In two brothers with autism and mild intellectual disability, Crepel and colleagues^53^ reported a 2 Mb duplication at 1q42.2: one breakpoint was within *KCNK1* and within the present association region, and the other breakpoint was just upstream of *DISC1*. In a study of expression differences in monozygotic twins discordant for bipolar disorder,^54^ *KCNK1* showed consistent overexpression in the affected twin. Conversely a meta-analysis by Mistry and colleagues^55^ found that the expression of *KCNK1* is reliably down-regulated in the prefrontal cortices of patients with schizophrenia. However, although 1q42.2 is a region that has been linked with psychotic illness^56^^,^^57^ and although the TWIK-1/TREK-1 heterodimer has been proposed as a target for antidepressant drugs,^58^ *KCNK1* is explicitly not among the loci that have been associated with schizophrenia by GWAS^59^^,^^60^: Indeed, in a 2014 GWAS,^56^ rs701232 had a thoroughly nonsignificant *P* value of 0.2832.

However, although *KCNK1* is clearly not itself a candidate gene for psychosis in clinical populations, we leave open the possibility that it is a route by which biochemical changes associated with psychosis can lead to alterations in ocular tracking—for example, via activation of cannabinoid receptor 1 (see above). In this context, we note the interesting finding by Sami et al*.*^61^ that patients with early psychosis who were heavy cannabis users did not exhibit the reduced gain in smooth pursuit that was seen in comparable patients who were not cannabis users.

### The Site of Action of *KCNK1*

The introduction of a covariate in GWAS may throw light on how a genetic polymorphism alters the phenotype. In the present study, the use of an independent phenotypic measure allowed us to constrain the probable site of action of *KCNK1*.

Individual differences in ocular tracking could arise from variation in the visual analysis of motion as well as from variation at different levels of the oculomotor system.^7^^,^^62^^–^^64^ Correlations between psychophysical judgements and oculomotor precision suggest that some of the variance in tracking ability indeed has its origin within the perceptual system. In a sample of 45 college students, Wilmer and Nakayama^7^ found that pre-saccadic pursuit acceleration correlated with psychophysical estimates of the speed of “low-level” motion, whereas the precision of post-saccadic pursuit correlated with judgments of “high-level” motion. In a sample of 36 healthy observers, Price and Blum^65^ found that the precision of perceptual judgements of motion direction was correlated with the precision of ocular tracking. For patients with schizophrenia, similar relationships have been found between the gain of smooth pursuit and psychophysical thresholds for detecting coherent motion^64^ and for discriminating velocity.^66^

For our own large population of young, healthy adults, phenotypic correlations of this kind are observed, and we exploited them to test whether the variance due to *KCNK1* is of perceptual origin. When we used as a covariate the ability to detect coherent motion in random noise,^43^ the association of smooth-pursuit RMSE with *KCNK1* was not weakened but instead slightly strengthened. This result suggests that the variance associated with chromosomal region 1q42.2 is unlikely to originate within the perceptual analysis of motion, but is more likely to originate in executive or motor processes—or possibly in the use of re-afferent information during the closed-loop phase of pursuit.^67^ If the variance derived from sensory mechanisms, we should have expected the association with rs701232 to become weaker when coherent motion sensitivity was used as a covariate. The subsidiary association with anticipatory saccades (often considered predictive^45^), but not with catch-up saccades, suggests a relatively central site for the action of TWIK-1.

### Sex Differences

Our study was not explicitly designed to study sex differences (to do so would require truly random sampling of males and females from the total parent population—something that is rarely achieved even in studies explicitly concerned with sex differences). In addition, in our cohort of volunteer participants there were more females than males, in the ratio 645:413. Thus it is conceivable that a sampling difference accounts for the sex differences we observe: our male and female participants may not have been equated with respect to some critical, but unidentified, factor.

However, our volunteers were drawn from a relatively homogeneous population of young adults in the Cambridge area (many of them were students from Cambridge University). Moreover, there is a further reason for placing these sex differences on record. The expression of *KCNK1* has been reported to be sex-dependent in other systems. In endomyocardial biopsies from patients with new-onset heart failure, *KCNK1* was overexpressed in males.^68^ In zone 3 of the mouse liver, phenobarbital leads to the induction of TWIK-1 in males but not in females.^69^

### TWIK-1 and pH

The ion channel TWIK-1 is pH dependent, and in a complicated way: At a pH of 7.4 the channel is open and is selective for K^+^ ions, but a reduction to a pH of 6 leads to the channel becoming less selective, so that an inflow of Na^+^ opposes the outflow of K^+^ and the net flow of positive charges is reduced.^70^^–^^72^ Is it possible that variation in pH, acting via TWIK-1, is the common pathway through which several factors affect smooth pursuit eye movements? We note the following: (i) Phenobarbital (but perhaps not all barbiturates) has been reported to reduce intracellular pH^73^; (ii) In *post mortem* brain tissue pH has been found to be lower in female than in male brains^74^ (although see reference 75); (iii) There are recurrent reports that pH is reduced in the brains of patients with schizophrenia^76^; and genes whose expression is associated with lowered pH are over-represented among the genes that are differentially expressed in schizophrenia and bipolar disorder.^77^ Each of these observations would be open to discussion; but we note the interesting possibility that variations in pH, acting via TWIK-1, offer a route by which several factors could affect smooth pursuit eye movements.

## Conclusions

We find no association between the precision of ocular tracking and three traditional candidate genes, *DRD3, COMT* and *NRG1*. Our results can be seen in the wider context of the frequent failure of GWAS to confirm candidate-gene studies.^78^^,^^79^ None of the three candidates has in fact proved to have a strong association with schizophrenia.^80^

Our study, however, does find a strong association of smooth pursuit performance and markers within the gene *KCNK1*, which encodes the two-pore-domain potassium channel TWIK-1 or K2P1. The effect is a large one (0.29 SD for each additional copy of the A allele). *KCNK1* is rendered a plausible candidate gene by the ion channel it encodes and by the known pharmacology of the channel. Limitations of our study are that our cohort is small by current standards of GWAS and that we do not have a replication cohort. Strengths of the study are the detailed phenotypic measurements, the explicit estimates of test-retest reliability, and the use of a perceptual covariate to narrow down the route by which *KCNK1* alters the phenotype.
